# Mind the Gap: The Implications of Not Acting in Line With Your Planned Actions After Installing Solar Photovoltaics

**DOI:** 10.3389/fpsyg.2019.01423

**Published:** 2019-06-26

**Authors:** Annemijn Maron Peters, Ellen van der Werff, Linda Steg

**Affiliations:** Environmental Psychology, Faculty of Social and Behavioural Sciences, University of Groningen, Groningen, Netherlands

**Keywords:** solar photovoltaic, self-identity, motivation, sustainable energy behavior, smart energy technologies

## Abstract

To realize the full potential of solar photovoltaics (PV), PV adopters need to adapt their energy demand to the production of self-generated solar energy as much as possible (i.e., use their PV sustainably). In a longitudinal questionnaire study (*N* = 74) in the Netherlands, we compared the intention to use PV in a sustainable way before the installation of PV with actual PV use. Wave 1 took place before respondents adopted PV, while Wave 2 took place after they installed PV. We examined whether potential differences between actual sustainable PV use and initial intentions may have implications for how people see themselves and for the motivation they ascribe to their decision to adopt PV. Our results show that the vast majority of people use their PV in a less sustainable way than they anticipated. Furthermore, after the installation of PV, respondents are less likely to see themselves as a sustainable PV user and less likely to believe that PV have positive environmental consequences than before the installation, while environmental self-identity did not differ pre and post-installation of PV. Moreover, the stronger the discrepancy between intended and actual sustainable use of the PV, the less likely people were to see themselves as a sustainable PV user and as a person who acts pro-environmentally in general. These findings suggest that it is important to support people to use their PV in a sustainable way to facilitate them to act upon their intentions.

## Introduction

The wide-scale adoption of smart energy technologies, such as solar photovoltaics (PV), is key to mitigating climate change. For example, PV reduce carbon emissions as they produce sustainable energy (Bahaj and James, 2007). Yet, PV are more likely to realize their full potential when people use them in a sustainable way, by matching their energy demand to the energy production of their PVs as much as possible (Geelen et al., 2013). We aim to study to what extent people plan to use PV in a sustainable way before they install PV, and whether their actual PV use is in line with their intended use. We further explore whether differences in intended and actual sustainable use of PV may have implications for how people see themselves and the motivation they ascribe to adopt PV; we elaborate on our reasoning below.

Actual use of PV may differ from intended use before the installation of PV. On the one hand, people may use their PV in a more sustainable way than they anticipated, as the presence of PV can make them more aware of the impact of their energy use on the environment, encouraging sustainable PV use (Keirstead, 2007; Kobus et al., 2013). On the other hand, people may use their PV in a less sustainable way than they intended, as adapting energy demand to the self-generated energy supply may prove more challenging than they anticipated (Schick and Gad, 2015; Schmalfuss et al., 2015; Smale et al., 2017).

The discrepancies between the expectation to use PV in a sustainable way and actual sustainable PV use might affect the perceptions people have about themselves. Specifically, we propose that people are likely to base their judgments about how sustainably they act by comparing their behavior to a reference point (cf. Tversky and Kahneman, 1974). The intention to engage in sustainable behavior may function as a reference point against which one’s current performance is judged. Moreover, people are motivated to be or to appear consistent and to align their cognitions, such as the way they see themselves, with their actions (Van der Werff et al., 2013b, 2014a, 2014b; Kashima et al., 2014; Steg, 2016). This consistency principle is a key element in various theories, such as self-perception theory that proposes that people infer their cognitions, such as perceptions about themselves, through observation of the behavior they engage in Bem (1972). Consistency is also a core principle in cognitive dissonance theory that assumes that incongruence between people’s cognitions and actual behavior may result in feelings of discomfort, and that people are motivated to reduce this discomfort by adjusting either their behavior, but particularly their cognitions as the latter is more easy to do (Festinger, 1957).

Based on these theories, we hypothesize that discrepancies between anticipated and actual sustainable PV use may affect the extent to which people see themselves as a sustainable PV user (i.e., their sustainable PV identity). When people use PV in a less sustainable way than intended, they are likely to see themselves as a less sustainable PV user; conversely, their sustainable PV identity may be strengthened when they use the PV in a more sustainable way than they anticipated. To our knowledge, it has not yet been tested whether any discrepancy between people’s intentions and their behavior influences the way people see themselves. We aim to address this issue in the current study.

The question remains whether a discrepancy between the intention to use PV in a sustainable way and actual sustainable PV use might affect the extent to which people see themselves as a person who acts pro-environmentally in general (i.e., their environmental self-identity; Van der Werff et al., 2013a, 2013b). Will people see themselves as a less pro-environmental person in general when they use their PV in a less sustainable way than anticipated; and will their environmental self-identity be strengthened when people use their PV in a more sustainable way than they anticipated? This could have far reaching implications for the likelihood of sustainable behaviors in the future, as a strong environmental self-identity promotes the engagement in wide ranging sustainable behaviors, while a weak environmental self-identity inhibits consistent sustainable actions (Van der Werff et al., 2013a, b, 2014a, 2014b; Peters et al., 2018).

Yet, environmental self-identity is particularly affected by previous (un)sustainable behavior when people realize that they engaged in many different (un)sustainable behaviors (Van der Werff et al., 2014a). Hence, it may be that discrepancies between intended and actual sustainable PV use will merely influence one’s identity related to this specific behavior (i.e., sustainable PV identity) and not one’s general environmental self-identity. To rule out the possibility that environmental self-identity changes because people change their level of engagement in other sustainable behavior at the same time as well, we will also monitor changes in the level of engagement in other sustainable behaviors. This way, we are able to examine whether environmental self-identity changes because people use PV in a more or less sustainable way than they anticipated.

A discrepancy between intended and actual use of PV may also affect the reasons people ascribe to their decision to adopt PV (i.e., adoption motivation) due the desire to be or to appear consistent (Van der Werff et al., 2013b, 2014a, 2014b; Kashima et al., 2014; Steg, 2016). Specifically, when people use their PV in a more sustainable way than anticipated, their sustainable PV identity and maybe even their environmental self-identity could be strengthened, which may make people think that environmental reasons played a more important role in their decision to adopt PV than they indicated beforehand. In contrast, when people use their PV in a less sustainable way than anticipated, they may perceive themselves as a less sustainable PV user and maybe even a less sustainable person in general, which may make them think that environmental reasons played a less important role in their decision to adopt PV than they indicated beforehand.

In sum, our aim was to examine whether there is a discrepancy between the intention to use PV in a sustainable way and actual PV use, and whether a difference between intended and actual use of PV may have implications for the way people see themselves and the motivation they ascribe to adopting PV. We hypothesized that people are more likely to see themselves as a sustainable PV user when their behavior (i.e., use of PV) is more sustainable than anticipated, while they are less likely to see themselves as a sustainable PV user when their behavior is less sustainable than anticipated. In addition, we will explore whether this discrepancy might even affect the extent to which people see themselves as someone acting environmentally friendly in general (i.e., environmental self-identity) and motives people ascribe to adopting PV (i.e., adoption motivation).

To test our reasoning, we conducted a longitudinal questionnaire study with two waves. Wave 1 took place before respondents adopted PV, while Wave 2 took place after they installed PV. We assessed the relevant variables (i.e., intention to use PV in a sustainable way or actual sustainable PV use, sustainable PV identity, environmental self-identity and environmental motivation to adopt PV) before (Wave 1) and after (Wave 2) people installed PV. As argued above, as environmental self-identity depends on the level of engagement in different types of sustainable behavior, not only the use of PV, we also measured the extent to which people engage in other sustainable behaviors both pre- and post-PV adoption.

## Materials and Methods

### Participants and Procedure

The study has been conducted in the North of the Netherlands. This region is currently facing earthquakes induced by gas drilling, which causes damage to property. As part as the risk mitigation and compensation program, house-owners who had at least €1.000 euro damage to their house resulting from gas drilling-induced earthquakes were eligible to apply for a subsidy to improve the energy efficiency of their houses to promote the sustainable energy transition, in addition to financial compensation to repair the damage. House-owners could apply for a subsidy up to €4.000 euro, which could be used for various investments such as installing double-glazing or PV. The organization assessing the subsidy applications invited people who applied for the subsidy to participate in our study via email.

**FIGURE 1:**
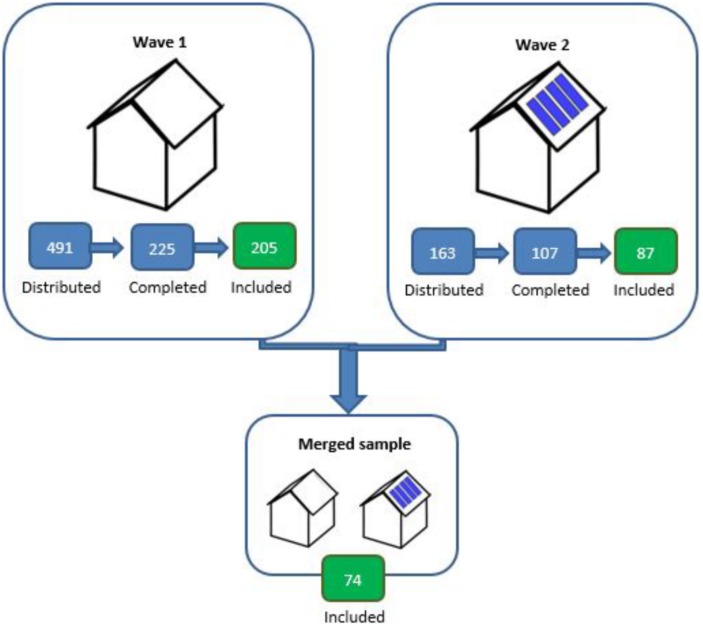
Flowchart of the distributed questionnaires, completed questionnaires, and questionnaires included in data analyses of Wave 1, Wave 2, and the merged sample.

We conducted a longitudinal questionnaire study with two waves. The first questionnaire (Wave 1) was administered after people completed the application for the subsidy to adopt PV (pre-PV adoption), between April 3–November 2, 2017. The second questionnaire (Wave 2) was sent after the PV had been installed to all participants who indicated in Wave 1 to be willing to participate in Wave 2, on November 21, 2017. In both waves, participants received a reminder to participate in the study approximately 2 weeks after the first invitation. In Wave 1, the questionnaire was sent to 491 people; 260 of them started the questionnaire, of which 225 completed the questionnaire (response rate of 45.8%). Of these, 20 participants indicated to have already installed PV. We did not include them in the data analyses, as we are interested in participants’ motivations and behavior before the installation of PV.^1^ The final sample in Wave 1 thus comprised 205 participants, of which 163 provided consent to be invited for participation in Wave 2 (see Figure 1). In Wave 2, 113 participants started the questionnaire; 107 participants completed the questionnaire, of which 20 did not install the PV yet. We did not include these 20 respondents in the data analyses, as we are interested in motivations and behaviors after the installation of PV.^2^ The final sample in Wave 2 thus consisted of 87 participants (Figure 1).

Appendix 1 provides an overview of the socio-demographic characteristics of the respondents. Compared to the general Dutch population, our sample comprised more men who were relatively older, living in larger households. Furthermore, the sample comprised relatively fewer very low or very highly educated people, and fewer people with a low income (CBS, 2018a, 2018b, 2018c).

To examine whether there is a discrepancy between the intention to use PV in a sustainable way and actual sustainable PV use, and to what extent any discrepancies between intended and actual PV use have implications for the way people see themselves and the motivation they ascribe to adopting PV, we only analyzed data from participants who participated in both Waves and who allowed us to link their data from Wave 1 and Wave 2 (*N* = 77). After merging Wave 1 and 2, three more participants were excluded from data analyses, as their data on socio-demographics showed that different people filled out the two questionnaires. Hence, the analyses were conducted including 74 participants^3^ (15 females, 58 males, one participant did not indicate gender; *M*_age_ = 59.47, *SD*_age_ = 11.23; Figure 1).

### Measures

The questionnaires of Wave 1 and 2 included the same questions, unless otherwise indicated^4^.

#### Sustainable PV Use

Respondents indicated to what extent they intended to adjust their energy use to match the self-generated PV energy supply (Wave 1), and to what extent they actually adjusted their energy use to the energy production of their PV (Wave 2). More specifically, in Wave 1 participants were asked to indicate on a 7-point scale, ranging from never (1) to always (7), to what extent they intend to engage in the following behaviors: primarily use electricity when the production by my PV system is high; use as little electricity as possible when the production by my PV system is low; using as little electricity as possible from the grid. In Wave 2, participants were asked to indicate how often they actually engaged in these behaviors. The items formed reliable scales, for both intention to use PV in a sustainable way (α = 0.70) and actual sustainable use of PV after its installation (α = 0.86); therefore the mean scores on the items were computed.

#### Sustainable PV Identity

Participants were asked to what extent the adoption of PV would make them a sustainable person. Specifically, participants indicated to what extent they agreed with the following item: the adoption of PV makes me a sustainable person; scores could range from totally disagree (1) to totally agree (7).

#### Environmental Self-Identity

Participants were asked what extent people see themselves as a person who acts pro-environmentally in general. Participants indicated on a 7-point scale, ranging from not at all (1) to certainly yes (7), to what extent they agreed with the following items: Acting pro-environmentally is an important part of who I am; I am the type of person who acts in an environmentally friendly way; I see myself as an environmentally friendly person (Van der Werff et al., 2013a, 2013b). The items formed a reliable scale (Cronbach’s alpha α = 0.87 in Wave 1, Wave 2 α = 0.88); hence mean scores were computed.

#### Sustainable Behaviors

Participants indicated on a 7-point scale, ranging from never (1) to always (7), how often they engage in twelve sustainable behaviors (see Appendix 2, based on Whitmarsh and O’Neill, 2010; Van der Werff et al., 2014a, 2014b; Peters et al., 2018).

#### Environmental Motivation to Adopt PV

We included two indicators of motivation to adopt PV: the extent to which PV is expected to have different environmental consequences and the importance of these consequences for the decision to adopt PV (cf. beliefs and evaluations, respectively, Ajzen and Fishbein, 2000). First, participants were asked to rate on a 7-point scale, ranging from totally disagree (1) to totally agree (7), to what extent they agreed that PV would have different environmental consequences (items are adapted from Noppers et al., 2014, 2015, 2016; Korcaj et al., 2015; Peters et al., 2018). We measured environmental PV beliefs with the following three items: PV reduce environmental problems, PV improve air quality, PV emit few greenhouse gasses. The items measuring environmental PV beliefs (Cronbach’s alpha α Wave 1 = 0.74; Wave 2 α = 0.81) formed a reliable scale; therefore the mean score of the items included in the scale was computed.

Second, participants indicated how important these environmental consequences of PV were for their decision to apply for a subsidy to adopt PV on a 7-point scale, ranging from very unimportant (1) to very important (7). The items measuring the importance of environmental consequences (Cronbach’s alpha α Wave 1 = 0.89, Wave 2 α = 0.89) (Cronbach’s alpha α Wave 1 = 0.80; Wave 2 α = 0.83) formed a reliable scale; therefore the mean scores of the items included in the scale were computed.

### Analyses

To examine whether sustainable PV use intention differed from actual sustainable PV use, and whether sustainable PV identity, environmental self-identity, sustainable behaviors, and environmental PV beliefs and evaluations differ before versus after the installation of PV, we conducted paired samples *t*-tests among participants who completed both Wave 1 and 2 (*N* = 74). To reduce the risk of Type I error due to multiple testing, we employed a Bonferroni corrected significance level of α (0.05/17) = 0.003. We computed effect sizes in two ways: using the standard deviation of the pre-test score (Cohen’s *d*^5^ ; Morris and DeShon, 2002) and pooled standard deviations of both pre- and post-test scores (Cohen’s *d* pooled; Dunlap et al., 1996). In both cases effect sizes are corrected for correlations between the means (Dunlap et al., 1996).

To examine whether any discrepancy between people’s intentions and their actual PV use is related to changes in their identity (i.e., sustainable PV identity and environmental self-identity) and motivations (i.e., environmental PV beliefs and motivations), regression analyses were conducted. More specifically, we regressed respondents’ sustainable PV identity in Wave 2 on the difference score between intention to use PV in a sustainable way and actual sustainable PV use, while we controlled for sustainable PV identity in Wave 1. Furthermore, we regressed respondents’ environmental self-identity in Wave 2 on the difference score between intention to use PV in a sustainable way and actual sustainable PV use, while we controlled for environmental self-identity in Wave 1 and changes in the extent to which people engaged in other sustainable behaviors. To conduct the latter analysis, we combined the other sustainable behaviors into one sustainable behavior index (Cronbach’s alpha α Wave 1 = 0.70; Wave 2 α = 0.73) and deducted Wave 2 from Wave 1. Lastly, we regressed people’s environmental PV beliefs and PV evaluations on the difference score between intention to use PV in a sustainable way and actual sustainable PV use, while controlling for the scores on environmental PV beliefs and evaluations in Wave 1.

## Results

### Differences in Sustainable PV Use, Identity, Sustainable Behaviors, and Environmental Motivations Before Versus After PV Adoption

The majority of participants used their PV in a less sustainable way than intended before the installation of PV. More specifically, 51 participants used the PV in a less sustainable way than they anticipated, while only 17 participants used the PV in a more sustainable way than they anticipated; the remaining four participants used the PV as they intended (2 missing).^6^

Analyses including the respondents that completed both Wave 1 and 2 (*N* = 74) showed that participants were more likely to see themselves as a sustainable PV user prior to installation (*M* = 5.41, *SD* = 1.25) than after installation of PV [*M* = 4.81, *SD* = 1.35; *t*(73) = 3.55, *p* = 0.001, *d* = −0.44; see Table 1]. Yet, environmental self-identity did not significantly differ before (*M* = 4.96, *SD* = 1.24) and after the installation of PV [*M* = 4.84, *SD* = 1.13; *t*(72) = 1.10, *p* = 0.267, *d* = −0.12; see Table 1]. In addition, no significant differences were found in the level of engagement in the twelve sustainable behaviors before versus after PV installation (see Table 2). Furthermore, after the installation of PV, participants less strongly believed that PV have positive environmental consequences (*M* = 5.55, *SD* = 1.16) compared to before the installation of PV [*M* = 6.04, *SD* = 1.01; *t*(71) = 3.42, *p* < 0.001, *d* = −0.43; see Table 1]. However, the extent to which participants evaluated these environmental consequences to be important in their decision to apply for the subsidy to install PV did not differ between before (*M* = 5.73, *SD* = 1.25) and after PV installation [*M* = 5.29, *SD* = 1.29; *t*(71) = 2.52, *p* = 0.014, *d* = −0.30; see Table 1].

**TABLE 1:** Differences in sustainable PV use intentions and behavior, and between beliefs, evaluations, sustainable PV identity, environmental self-identity, before versus after the installation of PV.

**Measure**	***N***	**Pre-installation**	**Post-installation**	**Difference in mean score^a^**	**95% CI**	***t***	***df***	***p***	**Cohen’s *d***	**Cohen’s *d* pooled**
								
		***M* (*SD*)**	***M* (*SD*)**	***M* (*SD*)^b^**						
PV intention (pre) and PV use (post installation)	72	4.37 (1.40)	3.32 (1.54)	1.05 (1.45)	(0.71; 1.39)	6.16	71	<0.001^*^	−0.77	−0.73
Beliefs about environmental consequences PV	72	6.04 (1.01)	5.55 (1.16)	0.49 (1.22)	(0.20; 0.78)	3.42	71	0.001^*^	−0.43	−0.40
Evaluations environmental consequences PV	72	5.73 (1.25)	5.29 (1.29)	0.44 (1.48)	(0.09; 0.79)	2.52	71	0.014	−0.30	−.30
Sustainable PV identity	74	5.41 (1.25)	4.81 (1.35)	0.59 (1.44)	(0.26; 0.93)	3.55	73	0.001^*^	−0.44	−0.42
Environmental self-identity	73	4.96 (1.24)	4.84 (1.13)	0.12 (0.96)	(−0.10; 0.35)	1.10	72	0.276	−0.12	−0.13

^*^p < 0.003 Bonferroni corrected. ^*a*^Wave 1–Wave 2; a positive difference score means that the mean score in Wave 2 was lower than the mean score in Wave 1. ^*b*^Due to rounding subtracting post-installation scores from pre-installation scores sometimes not exactly results in the difference mean scores provided in the table.

**TABLE 2:** Differences in sustainable behaviors before versus after the installation of PV.

**Measure**	***N***	**Pre-installation**	**Post-installation**	**Difference in mean score^a^**	**95% CI**	***t***	***df***	***p***	**Cohen’s *d***	**Cohen’s *d* pooled**
								
		***M* (*SD*)**	***M* (*SD*)**	***M* (*SD*)^b^**						
Turning the laptop/computer off when not in use, rather than leaving it on standby	73	5.38 (1.95)	5.21 (1.92)	0.18 (1.81)	(−0.24; 0.60)	0.84	72	0.402	−0.09	−0.10
Turning off the heating 1 h before I go to bed	72	5.57 (2.01)	5.29 (2.07)	0.28 (1.50)	(−0.08; 0.63)	1.57	71	0.121	−0.19	−0.19
Showering no longer than 3 min	73	4.00 (2.03)	4.04 (1.87)	−0.04 (1.65)	(−0.43; 0.34)	−0.21	72	0.832	0.02	0.02
Cycling short distances	73	5.82 (1.58)	5.42 (1.69)	0.40 (1.12)	(0.14; 0.66)	3.05	72	0.003	−0.37	−0.36
Turning off lights in rooms when no one is there	74	6.42 (1.02)	6.01 (1.14)	0.41 (1.20)	(0.13; 0.68)	2.90	73	0.005	−0.36	−0.34
Using public transport	73	2.64 (1.58)	2.55 (1.56)	0.10 (1.12)	(−0.17; 0.36)	0.73	72	0.467	−0.08	−0.08
Buying seasonal fruits and vegetables	74	5.11 (1.55)	5.07 (1.31)	0.04 (1.46)	(−0.30; 0.38)	0.24	73	0.811	−0.03	−0.03
Separating paper and glass from regular waste	74	6.69 (0.78)	6.68 (0.76)	0.01 (0.75)	(−0.16; 0.19)	0.16	73	0.877	−0.01	−0.01
Eating meat every dinner^c^	74	3.28 (1.46)	3.31 (1.48)	0.03 (0.95)	(−0.25; 0.19)	−0.25	73	0.807	0.03	0.03
Repairing items instead of throwing them away	74	4.86 (1.58)	4.70 (1.47)	0.16 (1.38)	(−0.16; 0.48)	1.01	73	0.314	−0.11	−0.12
Avoiding products with unnecessary packaging	73	3.18 (1.66)	3.23 (1.53)	−0.06 (1.72)	(−0.46; 0.35)	−0.27	72	0.786	0.03	0.03
Carpooling	73	1.58 (1.14)	1.81 (1.53)	−0.23 (1.15)	(−0.50; 0.04)	−1.73	72	0.088	0.25	0.21

^*^p < 0.003 Bonferroni corrected. ^*a*^Wave 1–Wave 2; a positive difference score means that the mean score in Wave 2 was lower than the mean score in Wave 1. ^*b*^Due to rounding subtracting post-installation scores from pre-installation scores sometimes not exactly results in the difference mean scores provided in the table. ^*c*^Reverse coded (the higher score, the less energy use).

### Relationships Between Difference in Intended and Actual Sustainable PV Use and Identity and Environmental Motivations in Wave 2

First, a regression analysis showed that sustainable PV identity in Wave 1 (β = 0.40, *t* = 3.67, *p* < 0.001) and the discrepancy between intended and actual sustainable PV use (β = −0.25, *t* = −2.28, *p* = 0.03) explained 18.1% of the variance in sustainable PV identity in Wave 2 [*F*(2,69) = 8.86, *p* < 0.001; see Table 3]. The less sustainable respondents used their PV compared to what they intended to do, the weaker their sustainable PV identity in Wave 2, when PV identity in Wave 1 is controlled for.

**TABLE 3:** Regression analyses to explain identity and motivations after the installation of PV.

	***R*^2^ adjusted**	***F***	***df***	**β**	***t***	***P***
**DV: Sustainable PV identity after the installation of PV**	0.18	8.86	2, 69			
Sustainable PV identity Wave 1				0.40	3.67	<0.001
Discrepancy intended and actual sustainable PV use				−0.25	−2.28	0.026
**DV: Environmental self-identity after the installation of PV**	0.55	29.74	3, 67			
Environmental self-identity Wave 1				0.72	8.93	<0.001
Change in the engagement in sustainable behaviors				−0.10	−1.25	0.214
Discrepancy intended and actual sustainable PV use				−0.22	−2.70	0.009
**DV: Environmental PV beliefs after the installation of PV**	0.12	5.63	2, 69			
Environmental PV beliefs Wave 1				0.37	3.34	0.001
Discrepancy intended and actual sustainable PV use				−0.03	−0.29	0.773
**DV: Environmental PV evaluations after the installation of PV**	0.09	4.66	2, 69			
Environmental PV evaluations Wave 1				0.31	2.75	0.008
Discrepancy intended and actual sustainable PV use				−0.13	−1.10	0.275

Second, a regression analysis showed that environmental self-identity in Wave 1 (β = 0.72, *t* = 8.93, *p* < 0.001), changes in the engagement in other sustainable behaviors (β = −1.02, *t* = −1.25, *p* = n.s.) and the discrepancy between intended and actual sustainable PV use (β = −0.22, *t* = −2.70, *p* = 0.009) explained 55.2% of the variance in environmental self-identity in Wave 2 [*F*(3,67) = 29.74, *p* < 0.001; see Table 3]. The less sustainable respondents used their PV compared to their intentions, the weaker their environmental self-identity in Wave 2, when environmental self-identity in Wave 1 and the difference in the engagement in other sustainable behaviors is controlled for.

The last two regression analyses showed that environmental PV beliefs and evaluations in Wave 2 were not significantly related to the difference score between intended and actual sustainable PV use, when controlling for environmental PV beliefs and evaluations, respectively, in Wave 1.

## Discussion

Smart energy technologies will only realize their full potential when people use them in a sustainable way, for example by adjusting one’s energy demand to match the self-generated energy as much as possible (Geelen et al., 2013). We conducted a longitudinal study to examine whether people use their PV in line with what they anticipated. We further examined whether differences in intended and actual sustainable use of PV may have implications for how people see themselves and for the motivation they ascribe to their adoption of PV.

Our results showed that the vast majority of participants used the PV in a less sustainable way than they anticipated before the installation of PV. Furthermore, as hypothesized, after the installation of PV, people are less likely to see themselves as a sustainable PV user (i.e., they have a weaker sustainable PV identity). Moreover, the stronger the discrepancy between intended and actual sustainable use of the PV, the less likely people were to see themselves as a sustainable PV user.

People’s general environmental self-identity and the extent to which they engage in other sustainable behaviors did not change after installing PV. Yet, interestingly, we found that the more respondents used their PV in a less sustainable way than they intended before the installation of PV, the weaker their environmental self-identity after the installation of PV. Future research is needed to study why environmental self self-identity did not change after installing PV, while the discrepancy between intended and actual sustainable use is related to environmental self-identity. This is important, as a strong environmental self-identity promotes the engagement in wide ranging sustainable behaviors, while a weak environmental self-identity may inhibit consistent sustainable actions (Van der Werff et al., 2013a, b, 2014a, 2014b; Peters et al., 2018). The finding that environmental self-identity did not change after installing PV may be due to the finding that people did not change the extent to which they engaged in other pro-environmental behaviors before versus after the adoption of PV. As such, our findings are in line with previous studies showing that environmental self-identity is relatively stable, and only changes when people realize that they engage in many different (un)sustainable behaviors, or behaviors that were relatively expensive and unique (Van der Werff et al., 2014a).

Our results suggest that after the installation of PV respondents were less likely to believe PV have positive environmental consequences than before the installation of PV (i.e., environmental PV beliefs). Yet, people’s evaluation of the importance of these environmental consequences of PV (i.e., environmental PV evaluations) in their decision to apply for the subsidy to install PV did not change. Furthermore, environmental PV beliefs and PV evaluations after the installation of PV were not significantly related to the discrepancy between intended and actual sustainable PV use.

In sum, the discrepancy between the intention to use PV in a sustainable way and actual sustainable PV use seem to particularly affect the way people perceive themselves, but not the motivation they ascribe to adopting PV. Our study is the first to show that a discrepancy between people’s intention to use PV in a sustainable way and people’s actual sustainable PV use may have implications for how people see themselves. These findings suggest that people indeed base their judgments about how sustainable they are by comparing their behavior to a reference point (cf. Tversky and Kahneman, 1974), in this case the intention to engage in sustainable behavior. Our results can also be explained by the motivation to be consistent (Cialdini, 1984; Guadagno and Cialdini, 2010), which implies that people are motivated to align the way they see themselves to their observed behavior. As such, our results are in line with theories that are based on the consistency principle. Specifically, our findings are in line with self-perception theory (Bem, 1972), showing that people adapt their cognitions (i.e., one’s sustainable PV identity and environmental self-identity) to their behavior (i.e., the extent to which they use their PV in a more or less sustainable way than intended). Similarly, our results are in line with cognitive dissonance theory (Festinger, 1957), which states that people are motivated to adjust their cognitions to their behavior. To our knowledge this is the first study to show that the way people perceive themselves is influenced by a discrepancy between intention and actual use of PV.

Future research could study why people used their PV in a less sustainable way than anticipated before the installation of PV. It may be that participants are not sufficiently motivated to adjust energy demand to match the production of self-generated solar energy, or that doing so is too costly in terms of effort or time. Indeed, studies suggest that sustainable use of smart energy technologies may prove more challenging than people anticipated (Schick and Gad, 2015; Schmalfuss et al., 2015; Smale et al., 2017).

Only a small minority used their PV in a more sustainable way than they intended, hence, we were not able to test what the implications of this difference between intended and actual sustainable use of PV might be for the way they see themselves and their motivations because the group size was too small. Future research could include larger samples including a larger group of people who uses the PV in a more sustainable way than expected to explore this issue in more detail.

Even though we started with a large number of participants (*N* = 491) and we achieved satisfactory response rates for both waves, we still ended up with a relatively small sample that completed the questionnaire in both waves (*N* = 74).^7^ This is typical in longitudinal studies. Moreover, our sample was self-selected and not fully representative of the Dutch population, which is not surprising as PV are still being adopted by a selective group of people that is not representative of the population. In fact, our sample was more representative for the Dutch population regarding income and education level than what is typically found in research on smart energy technology adoption (in which case male respondents with a relatively high income and education level are typically overrepresented, e.g., Rogers, 2010; Plötz et al., 2014). Yet, future research could test our reasoning among other samples to test the robustness of our findings.

Our study relied on self-reported data on motivation to adopt smart energy technologies, self-identity and anticipated and actual sustainable PV use and engagement in other sustainable behaviors. Obviously, measures of people’s motivations to adopt PV, identity and intended PV use typically rely on self-reports. Although self-reported behavior does not necessarily systematically differ from actual behavior (Gatersleben et al., 2002), future studies could include measures of actual use of self-generated solar energy, and use of energy from the regular grid. In addition, we included rather general measures such as “I primarily use electricity when the production by my PV system is high.” Future research could include more specific measures to examine PV use more precisely, for example, how often people use the washing machine, dishwasher or tumble dryer when their PV produce a lot of energy.

Our results suggest that it is important to empower and enable people to act upon their intention to use their PV in a sustainable way, as this may have consequences for how people see themselves. For example, specific guidelines can be provided on how people can adjust their energy demand to match the energy produced by their PV. Furthermore, people could be encouraged to adopt energy management systems, that automatically shift on or off household appliances, such as dishwashers or washing machines, depending on the self-generated energy production (Kobus et al., 2013; Sintov and Schultz, 2015). Household equipment can even be controlled and operated remotely, for example by distribution system operators. The question remains whether people are willing to adopt such appliances and programs, because people may be concerned about their privacy and autonomy (Sintov and Schultz, 2015). Future research is needed to examine how consumers can best be empowered to act upon their intention to use their PV in a sustainable way.

## Conclusion

To achieve a sustainable energy transition it is important that people not only adopt PV, but also use them in a sustainable way. We found that the vast majority of people used their PV in a less sustainable way than they anticipated. Moreover, after the installation of PV, people were less likely to see themselves as a sustainable PV user, but general environmental self-identity did not differ before and after the installation of PV. Furthermore, the stronger the discrepancy between intended and actual sustainable use of the PV, the less likely people were to see themselves as a sustainable PV user and as a person who acts sustainably in general. Moreover, respondents were less likely to believe PV have positive environmental consequences after the installation of PV than before the installation of PV. Yet, environmental beliefs after the installation of PV were not significantly related to the discrepancy between intended and actual sustainable PV use. These findings suggest that it is important to support people to use their PV in a sustainable way to facilitate them to act upon their intentions, as the discrepancy between intended and actual sustainable PV use seem to particularly affect the way people perceive themselves.

## Ethics Statement

The study was carried out in accordance with the recommendations of the National Code of Ethics for Research in the Social and Behavioural Sciences involving Human Participants as formulated by the National Ethics Council for Social and Behavioural Sciences with written informed consent from all subjects. The research protocol was approved by the Ethical Committee of Psychology of the Heymans Institute, University of Groningen.

## Author Contributions

AMP, EvdW, and LS designed the study. AMP collected and analyzed the data and drafted the manuscript. EvdW and LS were engaged in several rounds of revision of the manuscript.

## Conflict of Interest Statement

The authors declare that the research was conducted in the absence of any commercial or financial relationships that could be construed as a potential conflict of interest.

## Footnotes

1 In total, 171 participants did not fill in this question. We assumed they did not yet install PV, as the far majority in Wave 1 indicated that they did not know whether they would receive the subsidy.

2 We were not able to collect data among people who did not adopt PV. Our sample in Wave 2 included a small number of participants (*N* = 20) who did not install PV at that time, but they still planned to install PV.

3 We compared the key variables (i.e., environmental motivation to adopt PV, sustainable PV identity, environmental self-identity, intention to engage in sustainable PV use and intention to engage in other sustainable behaviors) of respondents who only completed the questionnaire in Wave 1 and respondents who completed the questionnaire both in Wave 1 and Wave 2. To reduce the risk of Type I error due to multiple testing, we employed a Bonferroni corrected significance level of α (0.05/17) = 0.003. The results showed no significant differences between both groups on the key variables. We also checked for differences in socio-demographics between respondents who only completed the questionnaire in Wave 1 and respondents who completed the questionnaire in both Wave 1 and Wave 2. To reduce the risk of Type I error due to multiple testing, we employed a Bonferroni corrected significance level of α (0.05/6) = 0.008. The results show that respondents who also completed the questionnaire in Wave 1 were somewhat older (*M* = 59, *SD* = 11.31) than respondents who only completed the questionnaire in Wave 1 [*M* = 54.49, *SD* = 11.07; *t*(192) = −2.73, *p* = 0.007]. The two groups did not differ on any of the other socio-demographics. This suggests that respondents who participated in both waves did not systematically differ from those who only participated in Wave 1.

4 Our questionnaire was part of a larger study. We only report the relevant items here; the full questionnaire is available upon request from the first author.

5 We reported only Cohen’s *d* in the text, Cohen’s *d* pooled can be found in Tables 1, 2.

6 Given the small numbers of participants that used their PV in an equal or more sustainable way than intended, we did not report differences in the dependent variables before and after the installations of PV for these groups. Follow up analyses show only a significant reduction in sustainable PV identity and environmental beliefs for those who used their PV in a less sustainable way than anticipated (*N* = 51), but not for those who used their PV in an equal (*N* = 4) or more sustainable (*N* = 17; 2 missing) way than anticipated. Yet, these findings should be interpreted with care given the small group sizes.

7 Yet, a power analysis with G^*^Power (Version 3.1.9.2, Faul et al., 2007) shows that a sample size of *N* = 74 is sufficient; to detect a medium effect of *d*_z_ = 0.5 with a 0.95 statistical power, *N* = 54 would be required.
