# Supplementary material for: Mind the Gap: The Implications of Not Acting in Line With Your Planned Actions After Installing Solar Photovoltaics
Source: Front Psychol. 2019 Jun 26;10:1423. doi: 10.3389/fpsyg.2019.01423 (PMC6606782; doi:10.3389/fpsyg.2019.01423)
Supplement: Supplementary file 1 [file Table_1.docx]

Appendix 1

Socio-demographic characteristics of respondents

|  | Wave 1^a^ | Wave 2^a^ |
| --- | --- | --- |
| **Gender** |  |  |
| Female | 51 (24.9%) | 23 (26.7%) |
| Male | 143 (69.8%) | 63 (72.2%) |
| Missing values | 11 (5.4%) | 1 (1.1%) |
| **Age** |  |  |
| *M* (*SD*) | 56.21 (11.35) | 59.03 (11.27) |
| Minimum | 30 | 32 |
| Maximum | 83 | 83 |
| Missing values | 11 | - |
| **Highest completed level of education** |  |  |
| Primary school | 1 (.5%) | - |
| Pre-vocational secondary education | 11 (5.4%) | 7 (8%) |
| Secondary vocational education | 62 (30.2%) | 32 (36.78%) |
| Senior general secondary education/pre-   university education/higher professional   education | 93 (45.4%) | 36 (41.4%) |
| University education | 21 (10.2%) | 11 (12.6%) |
| Other | 5 (2.4%) | 1 (1.1%) |
| Missing values | 12 (5.9%) | - |
| **Net income of one’s household per month** |  |  |
| Less than 1.000€ | 3 (1.5%) | 2 (2.3%) |
| Between 1.000€ - 2.000€ | 28 (13.7%) | 15 (17.2%) |
| Between 2.000€ - 3.000€ | 52 (25.4%) | 21 (24.1%) |
| Between 3.000€ - 4.000€ | 35 (17.1%) | 15 (17.2%) |
| Between 4.000€ - 5.000€ | 23 (11.2%) | 9 (10.3%) |
| More than 5.000€ | 14 (6.8%) | 8 (9.2%) |
| Not willing to indicate | 33 (16.1%) | 16 (18.4%) |
| Missing values | 17 (8.3%) | 1 (1.1%) |
| **Household composition** |  |  |
| Alone | 21 (10.2%) | 12 (13.8%) |
| With partner | 92 (44.9%) | 43 (49.4%) |
| With partner and child(ren) | 75 (36.6%) | 30 (34.5%) |
| With child(ren) | 6 (2.9%) | 2 (2.3%) |
| Missing values | 11 (5.4%) | - |
| **Number of people in the household** |  |  |
| 1 | 15 (7.3%) | 8 (9.2%) |
| 2 | 94 (45.9%) | 45 (51.7%) |
| 3 | 25 (12.2%) | 12 (13.8%) |
| 4 | 40 (19.5%) | 13 (14.9%) |
| 5 | 14 (6.8%) | 6 (6.9%) |
| Missing values | 17 (8.3%) | 3 (3.4%) |

**^a^** The percentages do not add up to 100, this is due to rounding

Appendix 2

Sustainable behavior items

|  |
| --- |
| 1. I turn my laptop or computer off when I do not use it instead of leaving it stand-by |
| 2. I turn the heating off one hour before I go to bed |
| 3. I do not shower more than 3 minutes |
| 4. I cycle short distances |
| 5. I turn off the lights in rooms when no one is there |
| 6. I carpool |
| 7. I use public transport |
| 8. I buy seasonal fruits and vegetables |
| 9. I separate paper and glass from regular waste |
| 10. I eat meat every dinner* |
| 11. I repair items instead of throwing them away |
| 12. I avoid products with unnecessary packaging |

*Note.* The following text preceded the items: “Please indicate how often you perform the following behaviors”. Answers were given on a 7-point scale, ranging from never (1) to always (7).
* Reverse coded (the higher score, the more environmentally-friendly)
